# RALGEF inhibitors suppress RAS-driven pancreatic cancer and metastasis

**DOI:** 10.1016/j.jbc.2025.110809

**Published:** 2025-10-08

**Authors:** Howard Donninger, Rachel Ferrill, Becca von Baby, Katie R. Hobbing, William L. Dean, Nagaraju Miriyala, Raphael Jigo, Joseph Burlison, John O. Trent, Robert Monsen, Geoffrey J. Clark

**Affiliations:** 1 Department of Medicine, University of Louisville, Louisville, Kentucky, USA; 2 Department of Pharmacology and Toxicology, University of Louisville, Louisville, Kentucky, USA; 3 University of Cincinnati College of Medicine, Cincinnati, Ohio, USA

**Keywords:** RAS protein, RALGDS inhibitor, pancreatic cancer, mitogen-activated protein kinase (MAPK), Akt, cell signaling

## Abstract

RAS oncoproteins are the most frequently activated oncoproteins in cancer. Development of direct RAS inhibitors has proved technically challenging and has had limited success in the clinic. Those RAS inhibitors that have been approved tend to suffer from resistance development. Consequently, many attempts have focused on inhibiting RAS indirectly by targeting its immediate downstream effectors. RAS binds and activates three main effector classes to drive transformation: RAF kinases, phosphoinositide 3 (PI-3) kinase and Ras-like (RAL) small GTPases (RALGEF) exchange factors. Multiple FDA-approved inhibitors for RAF and PI-3 kinase exist. So far, they have proved to be of limited effectiveness in patients. However, no inhibitors of the RALGEF effectors with demonstrated antitumor activity have been reported. This is despite the considerable body of evidence supporting a critical role for the RALGEF/RAL pathway in facilitating the *in vivo* transforming effects of activated RAS. Here, we describe the first small molecule pan-RALGEF inhibitor. We show the inhibitor specifically suppresses RAS/RAL signaling and exhibits antitumor effects in xenograft experiments, including a patient-derived xenograft (pdx) model. This *first-in-class* compound may lead to the development of more effective therapies for a broad range of RAS-driven tumors.

There are three closely related *ras* genes that produce four closely related RAS proteins, H, N, K-RAS4A and K-RAS4B. Mutation of a *ras* oncogene leading to constitutive activation of the RAS protein is one of the most frequent events in human cancer (1). A large body of evidence has been developed over the years to support the concept that these mutations are a key component of the development of malignant cancer (2, 3). Moreover, hyper-activation of non-mutant, wild-type RAS protein due to defects in its negative and positive regulators is also a common event in neoplasia (4). Indeed, some form of aberrant RAS activation may be a driving event in the majority of human cancers. Until recently, all attempts to develop a clinically effective RAS inhibitor had failed (5, 6).

As directly targeting RAS had proved so technically challenging, an alternative approach was adopted, targeting RAS function indirectly *via* its downstream effectors. RAS mediates its biological effects by binding and activating multiple downstream effector molecules which synergize to produce a net biological effect (7). The best-characterized are the RAFs, PI-3 kinase, and the RALGEF family of proteins. FDA approval has been granted for a number of RAF inhibitors and PI-3 kinase inhibitors to date, and more are in various stages of clinical trials (8, 9, 10). So far, these molecules have proved disappointing overall in the clinic (5). We hypothesize that this is in part because the most important RAS effector pathway for human cancer *in vivo* may actually be the third arm of the classic RAS effectors, the RALGEFs (11, 12, 13).

In 2021, the first direct RAS inhibitor was approved for use in lung cancer (14), followed recently by a second agent with a similar mechanism of action (15). These agents, Sotorasib (AMG510) and Adagrasib (MRTX849), only act on the K-RAS12C subset of RAS mutants. Yet, the approvals finally confirmed proof of principle for targeting RAS in human patients. More broad range direct RAS inhibitors have now been developed, some of which are now in clinical trials and appear promising (6, 16, 17, 18). However, although they can be very effective at suppressing the RAS/MAPK pathway, they appear much less effective at suppressing the RAS/RALGDS pathway (19).

The RAS/RALGEF/RAL pathway has been relatively neglected in RAS studies compared to the RAS/RAF and RAS/PI-3 kinase pathways. However, multiple studies have shown that the activation of the pathway, while unnecessary for normal growth of most cells in tissue culture, is essential for RAS driven transformation: Experimentally, the knockdown of RALA did not affect normal growth in 2D but severely suppressed the soft agar growth (3D growth) and xenograft tumor formation of 9/10 pancreatic carcinoma cell lines (13). Similar effects were found when the RALGEF family member RGL2 was inhibited by shRNA (20). In other studies, both RALA and RALB were essential for metastasis *in vivo* (21, 22). Moreover, RALGDS knockout mice resist RAS-induced transformation (23) and RALA knockout mice are extremely resistant to RAS-induced lung tumorigenesis (24).

There are four homologous members of the RAS effector RALGEF family (25), all containing consensus RAS Association (RA) domains in their C-terminus. The best-characterized members are RALGDS and RGL2 (also known as Rlf). RGL1 and RGL3 appear to be lower-affinity interactors with RAS. RALGEFs act as Guanine Nucleotide Exchange Factors (GEFs) for two RAS-related proteins called RALA and RALB. Thus, upon RAS activation, the RAL proteins become bound to GTP and activated *via* RALGEFS. The function of the RAL proteins remains only partially understood. When activated by RALGEFs, they bind and activate a series of effector proteins: RALBP1 (endocytosis and drug resistance), the exocyst components SEC5 and EXO84, FILAMIN, and the Y box transcription factor, ZONAB (11). They have also been shown to modulate the NF-kB pathway *via* TBK1 kinase (26). Thus, the RAL proteins modulate multiple biological pathways, several of which are vital for the development of tumorigenesis and metastasis (27, 28).

RAL proteins are usually found to be highly activated in pancreatic carcinoma cell lines, which are very frequently driven by K-RAS mutations (13, 20) and the activated RAL “transcription fingerprint” correlates with reduced survival in cancer (29). This may support the observation that RAS/RAL signaling promotes the development of cancer stem cells (30). Indeed, inducible knockdown of RALA in a primary pancreatic tumor model induced regression (21). Moreover, RALB suppression severely impairs metastasis in experimental pancreatic systems (21). Thus, the evidence is almost overwhelming that the RALGEF family of RAS effectors are key components of RAS-driven human cancer development and metastasis (13, 31, 32).

Here, we present the first small molecule pan-RALGEF inhibitor. We used a combination of molecular modeling and *in silico* library screening, followed by 3D *versus* 2D bioassays to identify a parental inhibitor compound with a 3D growth inhibition preference. The activity of this compound was enhanced by an iterative process of Medicinal Chemistry and bioassay. The resultant agent suppresses soft agar growth of RAS-driven tumor cells with an IC50 < 500 nM. It binds directly to RALGDS and impairs the interaction of RALGEFs with RAS. The most soluble derivatives show a low μM kd for binding to the RA domain of RALGDS. The compounds specifically suppress RAS signaling through the RAL pathway. We do not observe the same effect when we test the direct RAS inhibitors Sotorasib (AMG-510) or RMC-7977. The RALGEF inhibitor compounds exhibit no detectable toxicity *in vivo* but inhibit tumor formation and metastasis *in vivo*. We propose that this class of compounds may form the basis of a novel therapeutic approach to RAS-driven cancers and may cooperate with RAS inhibitors to fully suppress RAS pathways.

## Results

### Identification of RALGEF inhibitor

*In silico* screens were performed to identify chemical compounds with the potential to bind to the RA (RAS Association) domain of RALGDS in the conformation it adopts in complex with activated H-RAS (33). The top-ranked 96 compounds from the *in silico* screen were acquired from Molplex, whose libraries are pre-filtered for drug-like properties. Compounds were dissolved in DMSO for the assay.

**Figure 1:**
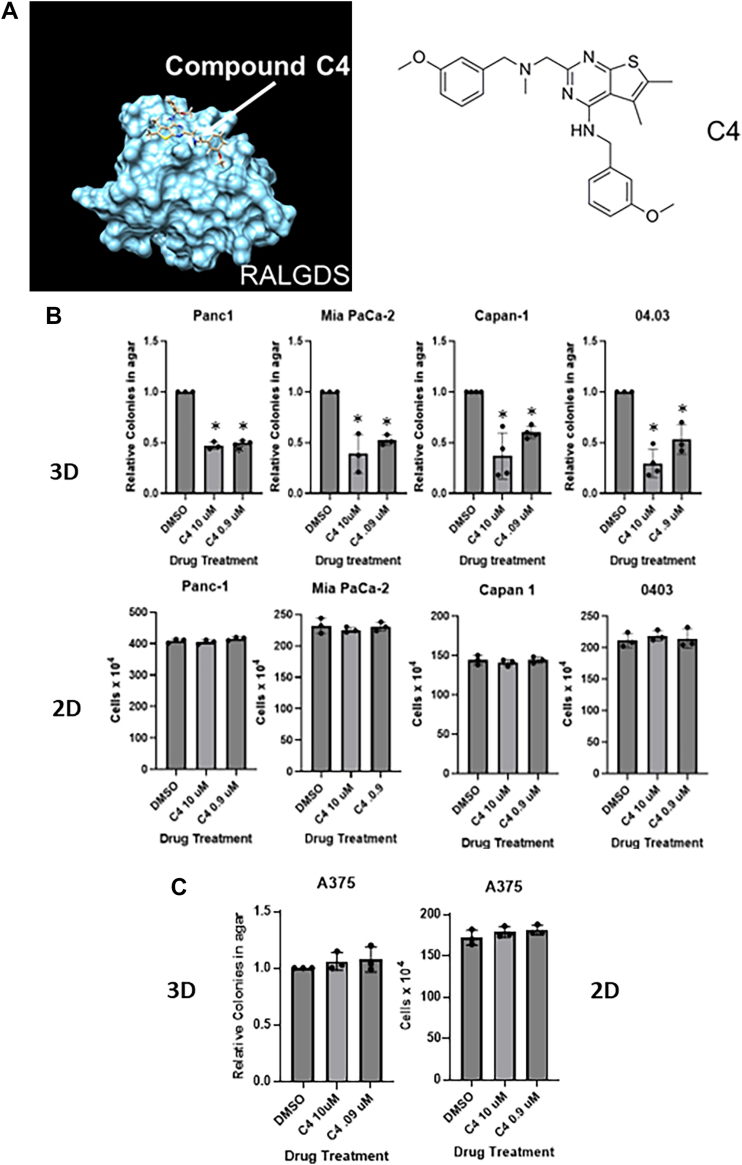
**C4/RALGDS complex and 3D vs 2D growth effects of C4.***A*, predicted interaction of parental molecule C4 in complex with RALGDS (*left panel*) and structure of C4 (*right panel*). (*B*) Effects of C4 RALGEF inhibitor on 3D soft agar growth (*upper panels*) and normal growth in 2D (*lower panels*) of Ras-driven pancreatic cancer cell lines PANC-1 (K-RAS12D mutant), MIA PaCa-2 (K-RAS12C mutant), Capan-1 (K-RAS12V mutant) and Panc 04.03 (K-RAS12D mutant). Colonies were counted 2 weeks after plating for the 3D assays, and cells were counted 1 week after plating for the 2D growth assays. (*C*) Similar experiments were performed on A375 cells, a B-RAF-driven melanoma cell line (wild-type RAS, B-RAF V600E mutant) as a control. Assays were repeated at least three times in duplicate and expressed as growth relative to the control. Analysis was by *t* test, error bars are standard error. ∗ *p* < 0.05.

Inhibition of the RALGDS/RAL pathway by shRNA blocks the ability of pancreatic tumor cell lines to grow in 3D soft agar assays but has no effect on the normal growth of the cell population in 2D tissue culture (20, 21, 34). Therefore, we screened the compounds in a 96-well format for the ability to suppress the growth in soft agar of a variety of RAS-driven pancreatic tumor cell lines, including MIA PaCa-2 and PANC-1. Several compounds exhibited activity. The most effective was designated C4. C4 is shown in predicted complex with RALGDS, binding to the RA domain (Fig. 1*A*).

Having identified C4, we then performed more quantitative assays focusing on mutant RAS containing PANC-1 and Panc 04.03 (KRAS12D), MIA PaCa-2 (KRAS12C), and Capan1 (KRAS12V) pancreatic tumor cells. A mutant B-RAF-driven melanoma cell line (A375) with wild-type RAS was used as a counter-screen control. We found that the compound gave IC50 values of less than 1 μM in the RAS-driven pancreatic cancer cell lines grown in 3D soft agar (Fig. 1*B*, top panels). One of the advantages of RALGEFs as targets is that their activation is essential for transformation, but not normal growth (20, 21, 34). Therefore, a RALGEF inhibitor should block agar growth but have little effect on normal 2D growth. When we assayed C4 against the same cell lines grown in 2D, we could detect no reduction in growth over the course of 1 week using the same concentrations that inhibited in 3D (Fig. 1*B*, lower panels). To confirm specificity to K-RAS, we then performed similar assays on the mutant B-RAF driven (wild type RAS) melanoma cell line A375 (Fig. 1*C*). Here, the same doses of the drug had no effect in 2D or 3D.

### Inhibition of RAS/RALGEF signaling by C4

**Figure 2:**
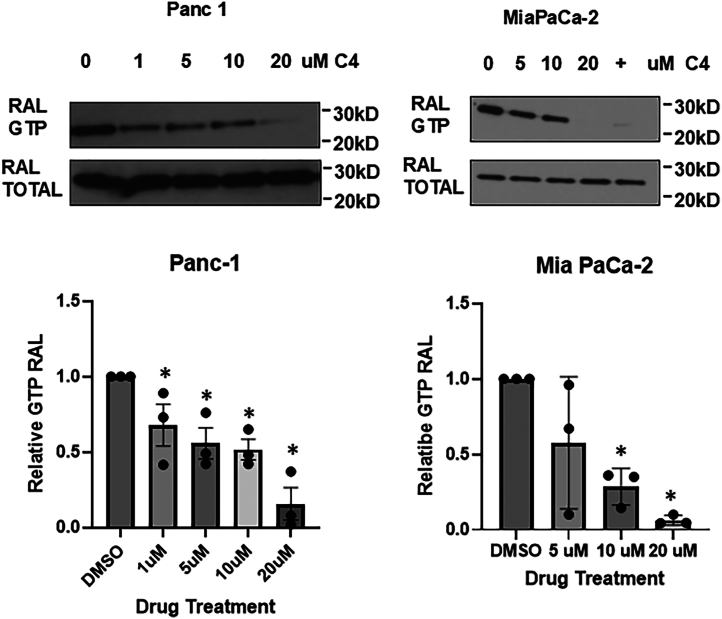
**C4 inhibits the RAL pathway in pancreatic cancer cells.** PANC-1 and MIA PaCa-2 cells were transiently treated (1 h) with the indicated concentrations of C4 and then lysed and assayed for the levels of active RALA using a commercial kit. The top panels show representative blots. + is an in-house RAS inhibitor included as a positive control. *Bottom panels* show the average of at least three separate assays. Analysis was by *t* test, error bars are standard error. ∗ *p* < 0.05.

RALGEFs drive RAL into the active, GTP bound form so it can bind its effector RALBP1. Inactive, GDP-bound RAL does not bind RALBP1. A commercial kit has been developed that uses RALBP1 attached to agarose beads as an affinity reagent specific to the active form of RAL. By measuring the level of activated RAL and comparing it to the total levels of RAL it is possible to measure the state of endogenous RAL activation. We assayed MIA PaCa-2 and PANC-1 cells containing endogenous mutant *ras* (K-RAS12C and K-RAS12D, respectively) for the effects of C4 treatment on endogenous levels of active RAL levels. Transient treatment (1 h) with C4 significantly suppressed the levels of endogenous active RAL (GTP bound) in both cell lines (Fig. 2).

### Identification of an enhanced activity derivative of C4

**Figure 3:**
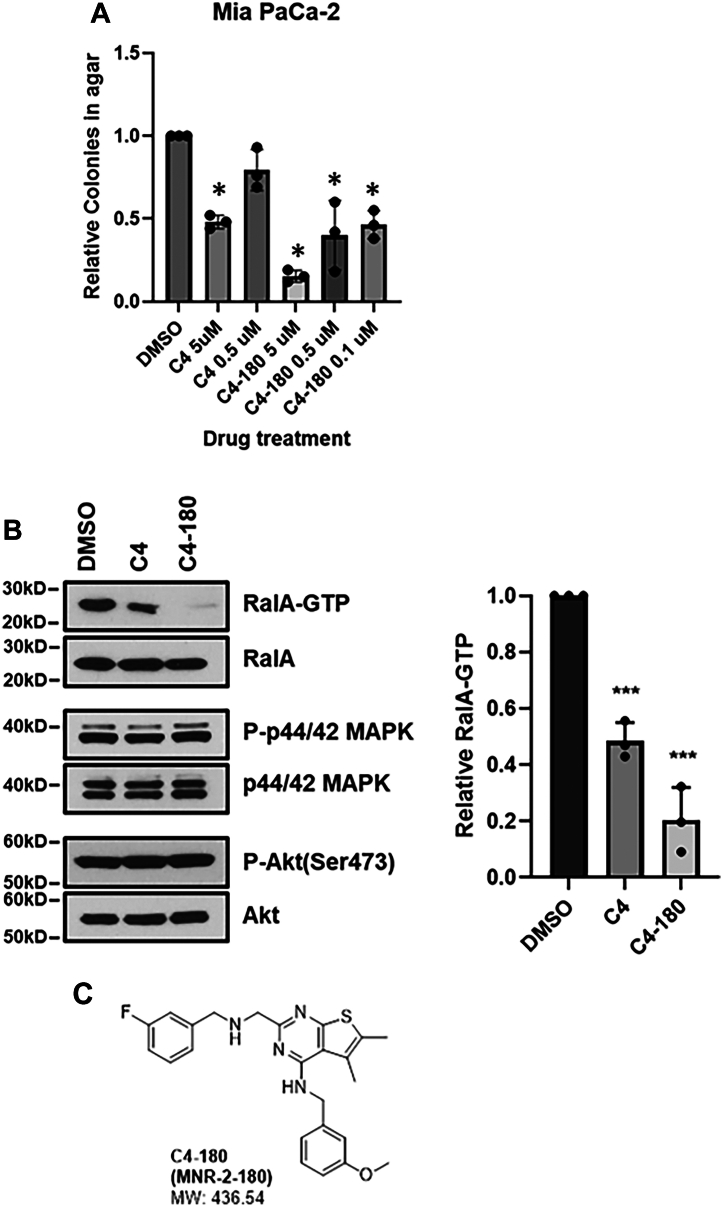
**C4-180, a derivative of C4 with enhanced activity specifically inhbits RAL but not MAPK/AKT signaling.** Medicinal chemistry was performed on C4 to generate structure-activity-relationships and a derivative, designated C4-180, was identified that showed enhanced activity over the parental compound C4. *A*, PANC-1 cells were seeded in soft agar and treated with the indicated doses of C4 and C4-180, and colonies counted 2 weeks after plating. A represenative of three duplicate experiments is shown; ∗ *p* < 0.05. *B*, PANC-1 cells were cultured in the presence of C4 and C4-180 (20 μM) for 1 h, lysed and the lysates analyzed by western blotting for RALA, MAPK and AKT pathway activation. Neither compound affected the levels of RAS/RAF/MAPK or RAS/PI3K/AKT signaling. Blot is representative of three independent experiments. Quantitation is shown on the *right* of the blot. Analysis was by *t* test, error bars are standard error. ∗∗∗*p* ≤ 0.005 relative to DMSO. *C*, structure of C4-180.

Multiple rounds of Medicinal Chemistry optimizaton (based on the Molecular Model) followed by soft agar screening were undertaken in an attempt to obtain an enhanced activity variant of C4. Over 170 derivatives of C4 were synthesized and tested against pancreatic cell lines in 3D soft agar assays. This led to the development of compound C4-180, which exhibits enhanced biological activity compared to the parental C4 compound (Fig. 3*A*). The effects of C4-180 on RAL activity were confirmed by RAL pull-down assay (Fig. 3*B*). C4-180 appears more active than C4 in suppressing RAL activity. The same lysates were examined for effects on the MAPK and PI3 kinase signaling pathways, and the compounds were found to be specific to RALGEFs as they did not affect the levels of phospho ERK or phospho AKT, respectively (Fig. 3*B*). The structure of C4-180 is shown in Figure 3*C*.

### Direct binding C4-180 with RALGDS

**Figure 4:**
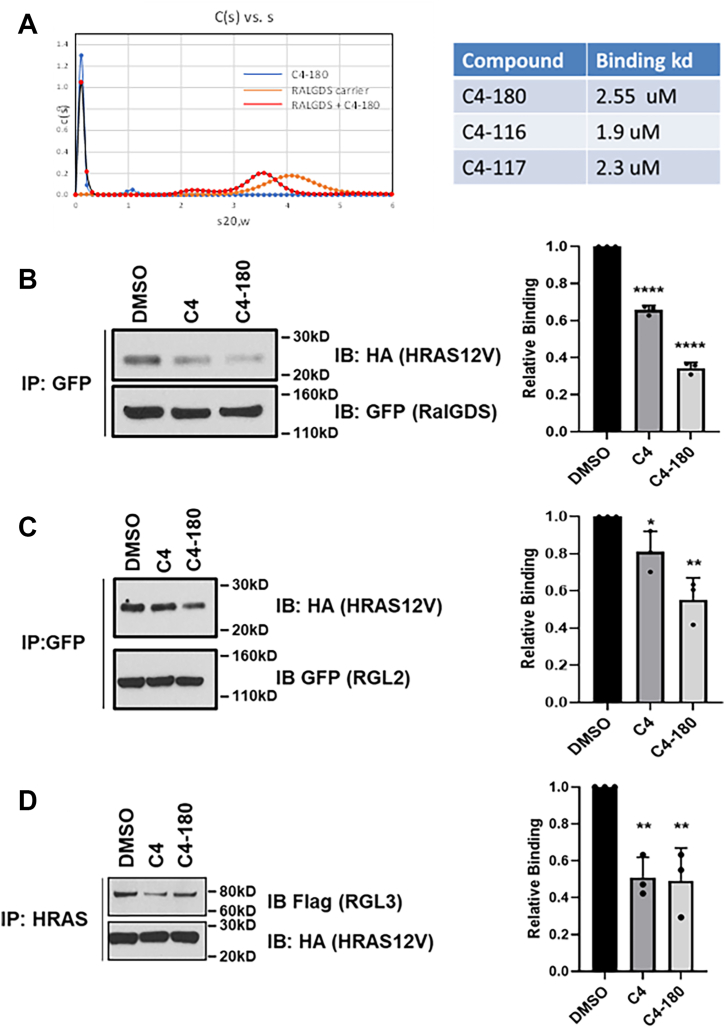
**C4-180 directly binds RALGDS and acts on both RALGDS and RGL2.***A*, analytical ultracentrifugation was used to show direct target binding of C4-180 to full-length RALGDS (*left panel*) and confirmed by MST assays (*right panel*) using the isolated RA domain of RALGDS and C4-180 plus several C4-180-related compounds. The latter compounds were significantly more soluble and exhibited better affinity for RALGDS. Values are the average of four experiments; the standard error was less than 50%. To confirm that the agents impair the ability of RALGDS to bind to RAS, lysates were prepared from HEK-293 cells expressing GFP-tagged RALGDS and HA-tagged H-RAS12V, mixed in the presence or absence of C4 or C4-180 (20 μM), immunoprecipitated with GFP beads and the immunoprecipitates analyzed by Western blotting for the presence of H-RAS and RALGDS (*B*). Similar experiments were performed with lysates from GFP-tagged RGL2- (*C*) and Flag-tagged RGL3- (*D*) expressing cells. Blots are representatives of 3 independent experiments, and quantitation is shown on the right-hand side. Analysis was by *t* test, error bars are standard error. ∗*p* ≤ 0.05, ∗∗*p* ≤ 0.01, ∗∗∗∗*p* ≤ 0.0001 relative to DMSO.

We performed target binding assays with C4-180 and the full-length RALGDS recombinant protein using Analytical Ultra Centrifugation (AUC). Figure 4*A* shows that C4-180 directly binds RALGDS with a predicted kd of ∼ 1uM. We then tested the compound against the isolated RA domain of RALGDS using Microscale Thermophoresis (MST), and obtained binding affinities in the low μM range. However, we felt solubility issues with C4-180 may have compromised this assay, and so we developed a new set of drivatives of C4-180 optimized for solubility. The most soluble of these (designated C4-116) gave an avarage kd of 1.9 μM in the MST assays (Fig. 4*A*, right panel).

### Inhibition of RAS/RALGEF binding

The anticipated mechansim of action of the compounds is to bind the RA domain of RALGDS and prevent it from interacting with RAS. However, the RA domain is conserved in the family suggesting a pan-RALGEF inhibitory activity. To confirm this, we expressed activated H-RAS, RALGDS as well as the related RGL2 and RGL3 separately in HEK-293 cells. We then lysed the cells and mixed the lysates in the presence or absence of compound. The lysates were then immunoprecipitated (IP) for RAS and western blotted (IB) for RALGEF to determine the degree of RAS/RALGEF complex present. The complex formation was suppressed. C4-180 was noticably better at suppressing the interaction of RAS with RALGDS and RGL2 than C4 (Fig. 4, *B* and *C*) but this effect was less obvious for RGL3 (Fig. 4*D*). We found the binding of RGL1 in similar studies too weak to quantify.

### Molecular modeling predicts how C4-180 binds RALGEFS and explains RALGEF RA domain specificity

**Figure 5:**
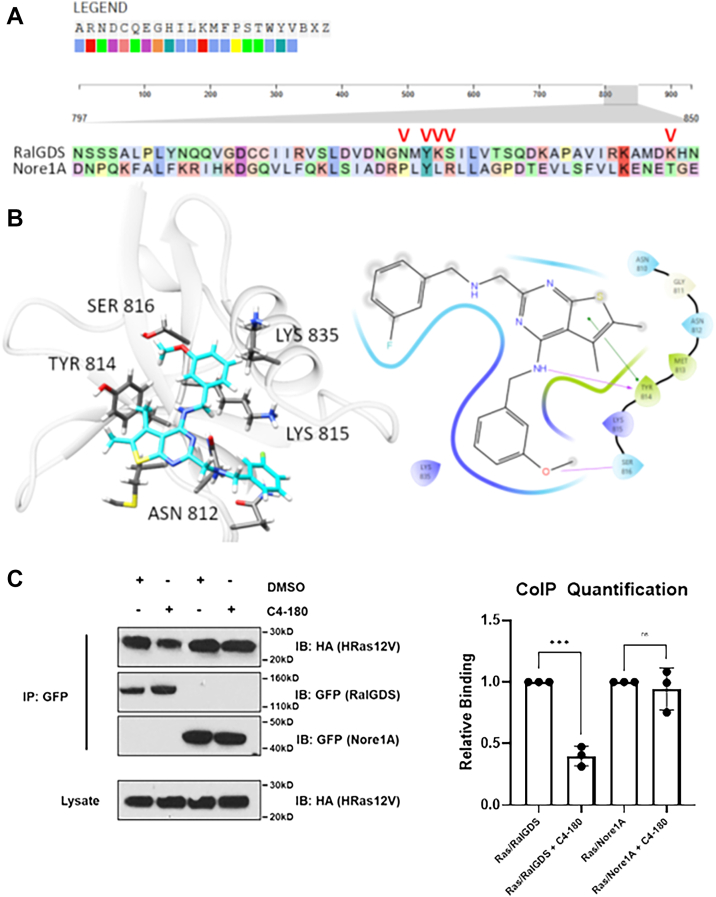
**The specific interaction of C4-180 with RALGEFs.***A*, Clustal Omega alignment of the Ras-associating domains of RASSF5 and RALGDS, showing low sequence similarity. Red carrot symbols indicate the highly conserved residues from RALGEF family members involved in their interaction with RAS. *B*, *left panel*: Compound C4-180 docked to the RALGDS-RAS binding interface using the GLIDE algorithm; *right panel*: Maestro ligand interaction diagram of C4-180 bound to RALGDS showing that major interactions are made with Tyr814 and Ser816 residues. *C*, co-immuno-precipitation studies of RAS with RALGDS and NORE1A (RASSF5), expressed in HEK-293 cells, in the presence or absence of C4-180 showing C4-180 is ineffective at blocking the interaction between RAS and NORE1A. *Left panel* is a representative blot; *right panel* shows quantitation of three experiments normalized to control. Analysis was by *t* test, error bars are standard error. ∗∗∗ *p* < 0.05. ns = not significant.

Clustal Omega alignment was performed on the RA domain of RALGDS in comparison to the RA domain of the RAS effector NORE1A (RASSF5) (Fig. 5*A*). This showed relatively low spatial conservation, particularly with the residues of RALGDS that are conserved with other RALGEF members known to be essential for binding to RAS (33). Docking C4-180 to the RALGDS RA domain using the GLIDE algorithm (Fig. 5*B*) predicts interactions with five major residues. Maestro ligand interaction (Schrödinger Release 2024-4: Maestro, Schrödinger, LLC, 2024.) prediction suggests that two of them (TYR814 and SER816) are the most important (Fig. 5*B*). These residues are conserved in the other RALGEF RA doimains but SER 816 is not conserved in the RA domain of the RAS effector NORE1A (RASSF5) (Fig. 5*A*). This explains the pan-RALGEF inhibiton effect and suggests the compound should not affect the interaction of RAS and NORE1A. To confirm the specificity, we repeated the RAS pull-down assays of Figure 4 using the full length NORE1A. C4-180 had little or no effect on the interaction of NORE1A with RAS (Fig. 5*C*).

### *In vivo* anti-tumor activity of C40-180

Transgenic knockout studies (23) and shRNA experiments on pancreatic cancer cells show that suppression of the RAS/RALGEF/RAL pathway inhibits the tumorigenic phenotype and the metastatic process, but does not have a severe impediment on normal cellular growth (20, 21, 34). To test the *in vivo* potential of C4-180, we performed xenograft experiments using different models of RAS transformation.

**Figure 6:**
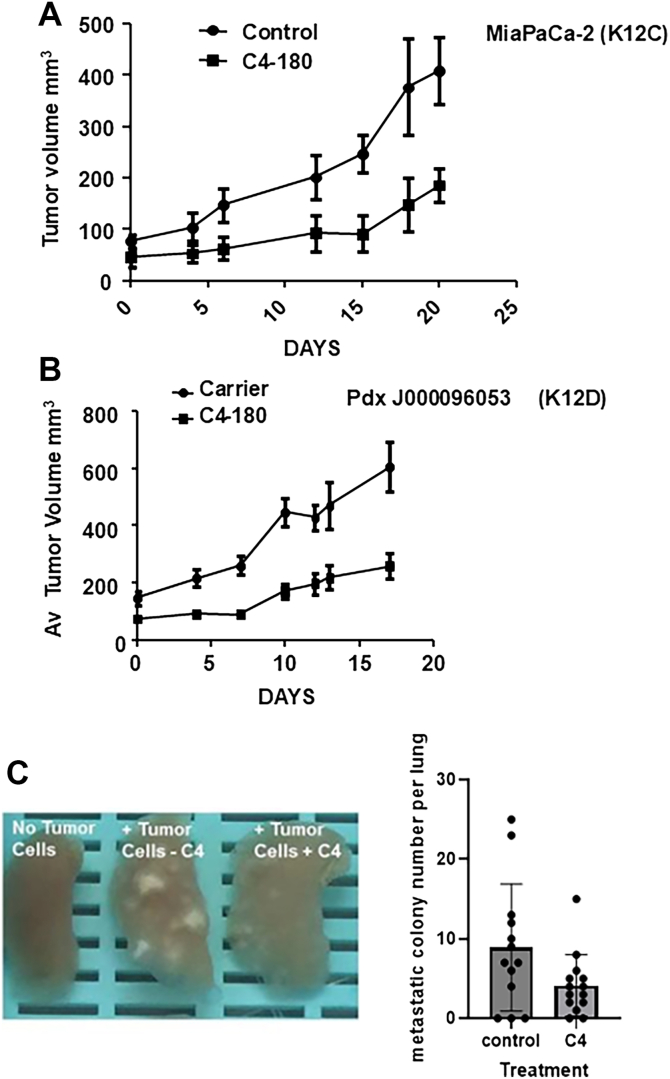
***In vivo* effects of C4/C4-180 on pancreatic cancer models.***A*, 1 × 10^6^ MIA PaCa-2 cells were subcutaneously injected into NSG mice (n = 6 per group). As tumors reached approximately 100 mm^3^ they were randomly assigned to treatment groups. Animals were injected with 40 mg/kg C4-180 on a 5 days on/2 days off cycle. Reduced tumor growth was found to be statistically significant (*p* = 0.038). Error bars are standard error, and two two-tailed *t* test using Graphpad Prism was employed to determine significance. *B*, effects of C4-180 on pancreatic pdx J0009653, which carries a K-RAS12D mutation. J0009653 pdx fragments were implanted into NSG mice (n = 5 per group, equal numbers of males and females). As tumors reached approximately 100 mm^3^ they were randomly assigned to treatment groups and were treated as described above in (*A*). C4-180 significantly reduced tumor growth (*p* = 0.0083). Error bars are standard error, and two tailed *t* test using Graphpad Prism was employed to determine significance. *C*, NRG mice were subcutaneously injected with 5 × 10^7^ MIA PaCa-2 cells near the abdominal fat pad region. On the day of injection, animals were randomized into two groups of 6 and administered (in a blinded fashion) C4 drug or carrier by i.p. injections. Injections were given at 10 mg/kg every other day for 5 days. After a further 2 weeks, the animals were euthanized, lung tissue slides were prepared and scored for metastatic colonies by a board-certified pathologist. *Left panel*, examples of lungs from treated and untreated mice; *right panel*, quantification of lung metastases number. Data were analyzed with Wilcoxon signed rank test, *p* = 0.0039.

MIA PaCa-2 cells are a pancreatic tumor cell line carrying the K-RAS12C mutation. Cells were injected into an equal number of NSG mice and as tumors arose, they were randomly assigned to experimental groups when they reached between 50 to 100 mm3 in volume. Animals were treated by i.p. injection with C4-180 daily 5-days on 2-days off, for 3 weeks and the tumor growth plotted. We observed a significant decrease in tumor growth in the mice treated with C4-180 compared to mice treated with the carrier (Fig. 6*A*).

Arguably the most stringent pre-clinical test of an anti-cancer drug is challenging against primary tumor xenograft (pdx). These systems have the most similarity of any model to a human tumor growing in a patient (35). PDX J000096053 carries the common K-RAS12D mutation. Animals were implanted with tumor grafts, and as tumors arose to between 100-200 mm^3^, they were randomly assigned to experimental groups for treatment. Animals were i.p. injected with 40 mg/kg for 18 days using a 5-day on, 2-day off schedule. Figure 6*B* shows that the RALGEF inhibitor treatment resulted in a statistically significant reduction of average tumor growth.

The most lethal component of cancer is metastasis. If sufficient MIA PaCa-2 cells are injected into the flank of NSG mice, we find that they can promote low levels of lung metastases. We injected mice, and the next day began a course of i.p. injections with C4 at 10 mg/kg daily for 10 days. After a further 2 weeks, we euthanized the animals and examined the lungs. Metastatic tumors were immediately obvious in many of the control animals (Fig. 6*C* left panel). Quantification of metastatic colony development was performed by scoring histopathological slides stained with H&E. We observed a statistically significant reduction in the numbers of metastatic colonies in the treated animals (Fig. 6*C*, right panel).

### Comparison of C4-180 efficacy against RAL activation to that of FDA-approved K-RAS 12C RAS inhibitor Sotorasib and pan-RAS inhibitor RMC-7977

**Figure 7:**
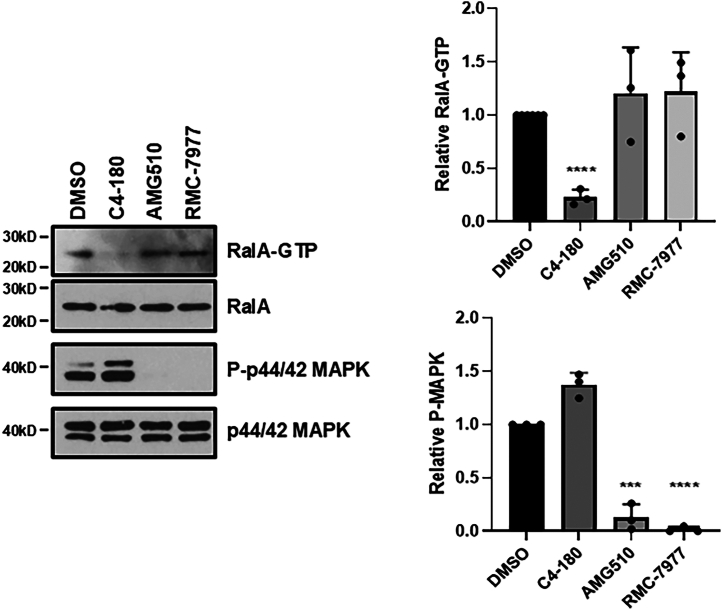
**Relative effects of C4-180 anti-RALGEF and commercial direct anti-RAS compounds on RAL activity.** MIA PaCa-2 cells were treated with the 20 μM of the indicated drugs for 1 h. Cells were lysed and equal amounts of lysates were analyzed for active RALA using an active RALA immunoprecipitation assay as well as MAPK activation by western blotting. A representative blot of three independent experiments is shown, and quantitation of the relative active RAL and MAPK levels is shown on the right of the blot. Analysis was by *t* test, error bars are standard error. ∗∗∗*p* ≤ 0.0005, ∗∗∗∗*p* ≤ 0.0001 relative to DMSO.

The approval of the direct KRAS12C inhibitor Sotorasib has been a seminal moment in the RAS field (36). This agent proves that direct inhbition of RAS can, in principal, be clinically effective. However, although Sotorasib (AMG-510) has potent inhibitory effects on the MAPK pathway it appears to provoke activation, rather than suppression, of the PI3K/AKT pathway (37). Its action on the RAL pathway has not been well studied. A pan-K-Ras inhibitor has been developed, but it too does not appear to act on the RAL pathway (19). The pan-RAS inhibitor RMC-7977 has a broader range, acting on all three main RAS proteins and all mutants of RAS (17). It is highly effective against the MAPK pathway but its effects on the RAL pathway are less clear. When we compared Sotorasib (AMG-510) and RMC-7977 to C4-180 in RAL activation assays in MIA PaCa-2 cells, we found they did not suppress RAL activation compared to C4-180, even though they completely abrogated MAPK pathway activation (Fig. 7).

## Discussion

RAS is the most frequently activated oncogene found in human cancer (1). Indeed, the mutation rate probably understates the importance of RAS deregulation, as upregulation of RAS activity due to defects in RAS regulating proteins such as NF1 and DAB2IP are also common in tumors (4). Developing a targeted therapy against RAS driven tumors has been something of a search for the Holy Grail for over 25 years. Until recently, direct inhibition of the RAS oncoproteins appeared to be impractical. This led to efforts to develop indirect inhibitors by blocking downstream components of RAS action. RAS activates three main mitogenic signaling pathways, the RAF/MAPK pathway, the PI-3K/AKT pathway and the RALGEF/RAL pathway. FDA-approved inhibitors of the RAF/MAPK pathway exist, as does an inhibitor of the PI-3 kinase pathway. Unfortunately, these compounds have proved of limited effect as single agents (38). Pre-clinical studies using combinations of the two-inhibitor classes have shown promising results (39), but it is unclear whether toxicity issues in patients will impede clinical use of this approach.

The RALGEF/RAL pathway makes up the third arm of the classic RAS signaling triumvirate. It is very much an understudied aspect of RAS biology. The PubMed database contains less than 5% of the publications on the RAL pathway as there are on the RAF/MAPK pathway. Our hypothesis was that chemically inhibiting RALGEFs could be an effective anti-RAS strategy. It was based on evidence from genetic modulation of the RAS/RALGEF/RAL signaling pathway in human systems (13, 21, 22, 31, 40) and knockout mouse studies (23, 24). These studies suggest that RAS/RALGEF signaling is essential to the development of the RAS-transformed phenotype, but not so important for normal cell growth. This could make RALGEFs an ideal target, as their inhibition may be intrinsically non-toxic. Recently, a covalent inhibitor specific for RGL2 was reported, but these studies did not include an examination of any cellular or *in vivo* activity (41). The lack of inhibitors with demonstrated biological activity for this class of molecule was an obvious deficiency in the anti-Ras armamentum.

Identifying small molecule inhibitors of the RAF/MAPK and PI-3 kinases was relatively straightforward, as they are kinases with ATP binding pockets. RALGEFs are a more challenging target, as there are no obvious classic pockets to target the protein/protein interaction (PPI) interface. However, our molecular modeling of the RALGDS RA domain in complex with activated RAS suggested that it might be practical. Testing of the *in silico* hits in *3D* *versus* *2D* growth inhibition assays allowed the identification of an inhibitor that mimicked the biological effects reported for genetic inactivation of RALGEFs inactivation. The inhibitor was far more potent in 3D soft agar assays than in normal 2D culture, as expected from the literature studies using genetic inhibition. The original inhibitor was designated C4, but Medicinal Chemistry enhancement of the initial compound led us to identify a more active agent, designated C4-180. This molecule had an IC50 in soft agar of < 500 nM. It also specifically suppressed the RAL signaling pathway in transient assays, as it had no detectable effect on the RAS/RAF/MAPK signaling pathway.

Confirmation of the direct target interaction proved difficult with the parental compound largely due to solubility issues. However, the C4-180 variant was found to be more soluble and showed binding to full-length recombinant RALGDS protein in AUC assays with an estimated kd of ∼ 1 μM. Solubility issues remained even with C4-180, and so a series of derivatives was generated that were specifically designed to enhance this property. In MST assays, these gave low μM binding affinities against the isolated RA domain of RALGDS. Analysis of the effects of the compounds on RAS/RALGEF complex formation showed that C4-180 could act on RALGDS, RGL2 and RGL3 family members. We also examined RGL1 and observed a similar effect, but the interaction with RAS was so weak that quantification was difficult. Thus, C4-180 appears to be the first pan-RALGEF inhibitor identified. Structural modeling of the docking of the compound to RALGDS explains this, as major predicted sites of interaction are conserved between the isoforms. However, they are not well conserved with the RA domain of the RAS effector NORE1A. When we tested NORE1A in RAS pull-down assays, we found that C4-180 had little or no effect on the complex formation. Thus, C40-180 is also specific for the RA domains of RALGEFs.

Xenograft experiments using the pancreatic cancer cell line MIA PaCa-2 showed that administration of the C4-180 derivative provoked a statistically significant reduction in tumor growth. This was only apparent in female mice. In males, while there was also a reduction in growth, it was not statistically significant. We suspect that this effect is due to the more aggressive growth of MIA PaCa-2 cells we observed in the male mice, perhaps because it is a male cell line. MIA PaCa-2 have previously been shown to be sensitive to sex hormone levels *in vivo* (42). Further *in vivo* studies used a RAS-driven pancreatic cancer pdx system. These systems are theoretically much more physiological than the cell line. Again, we were able to produce statistically significant reductions in tumor growth with C4-180. We did not observe sex-based differences in this system.

Metastasis is the main killer in cancer, and the RAL pathway has repeatedly been implicated as being essential in the metastatic program (43). We were also able to inhibit the ability of pancreatic tumor cells to metastasize to the lung. The experiment involved metastasis from a subcutaneous injection site, and so the whole metastatic program is required for successful metastasis, unlike the more common tail vein injection assay technique. We did not observe gender specific effects. The exact stage/stages of metastasis that were affected remains to be determined.

While we were developing these RALGEF inhibitors, molecules that directly bind to RAL proteins were identified and characterized (43). These are of considerable interest, although it appears our agents, operating upstream of them, are effective at lower doses. In addition, RALGEFs appear to have some poorly characterized oncogenic functions independent of activating RAL proteins (44) which we may also be compromising. Therefore, we have developed a first-in-class pan-RALGEF inhibitor that demonstrates *in vivo* antitumor activity.

With the approval of the covalent direct Ras inhibitor Sotorasib in 2021 (36), followed by Adagrasib (15) success with direct RAS inhibition in the clinic was finally achieved. However, these agents are specific to the KRAS12C variant only and the emergence of tumor resistance is common and quite swift (45, 46). Numerous other direct RAS inhibitors with broader action are now in various stages of clinical trials (6). There is considerable optimism that some of these may prove much more effective. However, in our hands, agents such as AMG-510 (Sotorasib), BI-2865 (pan-K-RAS) and RMC-7977 (pan-RAS) do not work well, if at all, on the RALGDS pathway. Similar results have been reported in the literature (19). The mechanisms for this remain unclear but could involve feedback loops or signaling from RAS-related proteins such as M-RAS or R-RAS2. Therefore, combinations of RALGEF inhibitors with the new RAS inhibitors may prove useful. This is an avenue we are actively investigating.

## Conclusion

Here we present the first pan-RALGEF oncoprotein inhibitor. The compounds suppress 3D *in vitro* cell growth, *in vivo* tumorigenesis and metastasis without obvious toxicity, as was predicted by previous genetic studies. Thus, the RALGDS pathway is a valid and targetable therapeutic avenue. There are now commercial inhibitors of RAS that are active against tumors in the clinic. These agents suffer from the problem of common, rapid resistance development. In our experimental systems, these agents do not work well against the RALGDS pathway. Thus, RALGDS inhibitors may be useful in combination therapy to suppress resistance development.

## Experimental procedures

### *In silico* screening

The RAS Association Domain (RA) of RALGDS (protein databank entry: 1LFD) was targeted by virtual screening using SurflexDock and the Molplex “in stock” library containing 1,300,000 compounds (3,194,093 ionic and tautomeric forms generated by LigPrep in the Schrodinger Suite). The protomol was generated using the RALGDS contact RAS residues with a proto_thresh of 0.18. The virtual screen was completed with the argument multi-start 5, results ranked, and the top 500 were output.

### Chemicals

The top 100 chemicals from the *in silico* screen were purchased from Molport and were dissolved in dimethyl sulfoxide (DMSO) as a carrier. Medicinal Chemistry was performed by the Institutional core facility. Chemical variants of the best candidate were generated in-house by the Institutional Medicinal Chemistry core. AMG-510 and RMC-7977 were purchased from Selleckchem.

### Tissue culture

Cell lines MIA PaCa-2 (K-RAS 12C) PANC-1 (K-RAS 12D), Capan1 (K-RAS12V), Panc 04.03 (K-RAS 12D) A375 (B-RAF V600E) and HEK-293 were purchased from the ATCC and used within 6 months of acquisition. MIA PaCa-2, PANC-1, A375 and HEK-293 cells were grown in Dulbecco’s modified Eagle’s medium (DMEM) supplemented with 10% fetal calf serum (FCS) (Corning), Capan1 cells were grown in DMEM supplemented with 20% FCS and Panc 04.03 cells were cultured in RPMI supplemented with 10% FCS. 2D growth assays were performed by seeding cells at low density (1 × 10^5^ cells) in 60 mm plates, in the presence or absence of drug. After 1 week, cell numbers were counted by hemocytometer. Soft agar assays were performed essentially as described (47). A bottom layer of 0.8% agar was plated and allowed to set, cells were diluted to 5000 cells per well and then plated in top agar (0.6%) in the presence or absence of the appropriate drug concentration. Assays were incubated for 2 weeks prior to scoring with an inverted microscope. Initial screens were performed in 96 well plates at a dose of 10 μM. Once potential candidates were identified, they were assayed in a more quantifiable 12 well format. Colonies with more than 10 cells were considered positive. Compound C4 was found to be the best initial hit.

### RAS/RALGEF binding assays

HA tagged activated H-RAS12V expression plasmid (48) and GFP-tagged full length RALGDS or RGL2 expression constructs (generous gifts, CJ Der) and RGL3 and RGL1 expression constructs (purchased from Genscript) were transfected separately into HEK-293 cells. Lysates were prepared the following day in modified RIPA buffer (150 mM NaCl, 50 mM Tris pH 7.5, 1% NP-40). Equal amounts of lysate were then mixed in the presence of carrier (DMSO) or anti-RALGEF compound at 10 μM and rotated for 1 h at room temperature. Samples were then immunoprecipitated with GFP beads (Allele Biotechnology). Pellets were washed three times before being assayed by Western analysis for the presence of HA-tagged RAS12V and GFP-tagged RALGDS, RGL2 and NORE1A, or Flag-tagged RGL3. GFP antibodies were purchased from Santa Cruz Biotechnology Inc, HA antibodies were purchased from Covance and Flag antibodies from Sigma. Secondary antibodies were purchased from Cell Signaling Technology.

### RAS/RALGEF signaling assays

The effects of the compound on the levels of active RAL were determined using a commercial RAL Activation Assay kit (#17-300) for active RAL (Millipore), as recommended by the manufacturer. Essentially, cells were grown to 70% confluence and then exposed to C4/C4-180 for 1 h. The cells were lysed, active RAL pulled down with a GST-RALBP1-RBD agent, and then the levels of active RALA to total RALA were measured by Western analysis to give a ratio of activation.

### MAPK pathway assays

Activation of the MAPK pathway in the same treated cells was performed by Western analysis of lysates balanced for total protein using phospho-ERK and ERK antibodies (#9101 and #9102, Cell Signaling). The same lysates were examined for PI3K pathway activation by using anti-phospho AKT and AKT antibodies (#4060 and #9272).

### *Direct RALGDS binding* assays

We used Medicinal Chemistry to generate a more soluble variant of C4 designated C4-180. We then used analytical centrifugation assays (AUC) to confirm a direct interaction of C4-180 with purified full length RALGDS protein (Genscript Biotech, NJ), essentially as described in (49, 50). We also measured binding affinity of C4-180 and several enhanced solubility derivatives to the isolated RALGDS RA domain using Microscale Thermophoresis (MST). MST traces were generated with a Monolith Nanotemper instrument using its MO. Control v2.0.4 software. Titration solutions consisted of a final concentration of 50 nM fluorescently labeled RALGDS mixed in a 16-point serial dilution series of compounds from 250 μM to 7.63 nM. Due to solubility issues of compounds, a background of 5% DMSO was required across all conditions. Dilutions were made in 384 well plates at a volume of 20 μl and incubated in the dark for at least 10 min before beginning MST analysis using standard capillaries. MST power was set to 40% in all cases. Data analysis was performed in the Nanotemper MO. Affinity Analysis software using a one-site binding model.

### Cell line xenografts

1 × 10^6^ MIA PaCa-2 pancreatic cancer cells (carrying a K-RAS12C mutation) were subcutaneously injected into female NSG mice (the tumors grew more aggressively in the male mice and showed less inhibition). When a tumor reached 50-100 mm^3^ it was randomly assigned to an experimental group (n = 6) and treated with C4-180 at 40 mg/kg 5 days/week (5 on two off) by i.p. injection for approximately 2 weeks. Tumor volumes were measured with calipers.

### Cell line metastasis assays

Animals were subcutaneously injected with 5 × 10^7^ MIA PaCa-2 cells. The day after injection, animals were randomized into two groups of 6 (equal mix of male and female) and given (blinded) i.p. injections of drug or carrier. Injections were given at 10 mg/kg every other day for 5 days. After a further 2 weeks, the animals were sacrificed, lung tissue slides prepared and scored for metastatic colonies by a board-certified pathologist. The experiment was performed twice independently.

### Pdx xenografts

Pancreatic pdx, J000096053 is derived from a K-RAS12D carrying tumor. pdx were obtained from Jackson Labs, Bar Harbor Maine. Animals were implanted with tumor fragments, and when tumors reached 100 to 200 mm³, they were randomly assigned to an experimental group (n = at least 6) and treated with either C4-180 at 40 mg/kg or carrier 5 days/week (5 on two off) by i.p. injection for approximately 3 weeks. Tumor volumes were measured with callipers.

All animal studies were pre-approved by the University of Louisville Institutional Animal Care and Use Committee (IACUC). Tumors were not allowed to exceed the maximum permitted size (1 cm^3^).

## Data availability

The data used to support the findings of this study are contained within the manuscript.

## Conflict of interests

The authors declare the following financial interests/personal relationships which may be considered as potential competing interests: GJC, JB and JOT are inventors on a patent on the inhibitors.
